# A mixed methods case study investigating how randomised controlled trials (RCTs) are reported, understood and interpreted in practice

**DOI:** 10.1186/s12874-020-01009-8

**Published:** 2020-05-12

**Authors:** Ben E. Byrne, Leila Rooshenas, Helen S. Lambert, Jane M. Blazeby

**Affiliations:** 1 Centre for Surgical Research, Population Health Sciences, Bristol Medical School, University of Bristol, Canynge Hall, 39 Whatley Road, Clifton, Bristol, BS8 2PS UK; 2 Population Health Sciences, Bristol Medical School, University of Bristol, Bristol, UK; 3 MRC ConDuCT-II Hub, Bristol Medical School, University of Bristol, Bristol, UK; 4 NIHR Bristol Biomedical Research Centre, University Hospitals Bristol NHS Foundation Trust, Bristol, UK

**Keywords:** Randomised controlled trial, surgery, Methods, Translational medical research, Health services research, Evidence-based medicine

## Abstract

**Background:**

While randomised controlled trials (RCTs) provide high-quality evidence to guide practice, much routine care is not based upon available RCTs. This disconnect between evidence and practice is not sufficiently well understood. This case study explores this relationship using a novel approach. Better understanding may improve trial design, conduct, reporting and implementation, helping patients benefit from the best available evidence.

**Methods:**

We employed a case-study approach, comprising mixed methods to examine the case of interest: the primary outcome paper of a surgical RCT (the TIME trial). Letters and editorials citing the TIME trial’s primary report underwent qualitative thematic analysis, and the RCT was critically appraised using validated tools. These analyses were compared to provide insight into how the TIME trial findings were interpreted and appraised by the clinical community.

**Results:**

23 letters and editorials were studied. Most authorship included at least one academic (20/23) and one surgeon (21/23). Authors identified wide-ranging issues including confounding variables or outcome selection. Clear descriptions of bias or generalisability were lacking. Structured appraisal identified risks of bias. Non-RCT evidence was less critically appraised. Authors reached varying conclusions about the trial without consistent justification. Authors discussed aspects of internal and external validity covered by appraisal tools but did not use these methodological terms in their articles.

**Conclusions:**

This novel method for examining interpretation of an RCT in the clinical community showed that published responses identified limited issues with trial design. Responses did not provide coherent rationales for accepting (or not) trial results. Findings may suggest that authors lacked skills in appraisal of RCT design and conduct. Multiple case studies with cross-case analysis of other trials are needed.

## Background

It is widely recognised that clinical practice is often not in line with the best available evidence. This is the so-called ‘gap’ between research and practice [1, 2]. Best evidence predominantly comes from well designed and conducted randomised controlled trials (RCTs) [3]. However, RCTs are often complex and challenging. Surgical RCTs present specific issues with recruitment, blinding of patients and surgeons, and intervention standardisation [4]. Many of these issues have been clarified with methodological research [5–10]. Such work has led to improvements in trial quality over time [11, 12]. However, the gap between trials and implementation of their results in practice persists [13], potentially compromising patient care and wasting resources. Reasons for the disconnect are myriad.

Trial findings that report putative evidence for a change in clinical practice may not be implemented because of poor conduct and reporting [14], limitations in generalisation and applicability [15], cost, and unacceptability of new interventions. Clinical culture may emphasise the importance of experience over evidence [16], and some clinicians may have limited numeracy skills required to understand and apply quantitative results from trials [17]. Appropriate understanding of RCTs is critical to implementation and of vital importance to clinicians, researchers and funders. We have previously described a novel approach to explore understanding and interpretation of RCT evidence, by examining writings about individual surgical trials [18]. The present study aims to apply this new method to a single case study: the TIME (Traditional Invasive versus Minimally invasive Esophagectomy) RCT [19]. The purpose is to better understand how this trial has been interpreted and to illustrate the potential of this novel approach.

## Methods

The methodology used in this study has been described in detail elsewhere [18] and will be summarised here. The approach represents a form of case-study research, comprising mixed methods analysis of documentary evidence relating to a published RCT [20]. Case-study approaches have been defined in various ways and used across numerous disciplines. Their central tenet is to explore an event or phenomenon in depth and in its natural context [21]. The ‘real-world context’ in this study was the landscape of published articles that interpreted, appraised and discussed implementation of the TIME trial’s findings. Our approach aligned with Stake’s ‘instrumental case-study’ [22], using a particular case (the TIME RCT’s outcomes paper) to gain a broader appreciation of the issue or phenomenon of interest (in this case, interpretation and appraisal of RCTs in the clinical community, and implications for implementation). We conducted qualitative analysis of selected published articles citing this RCT’s primary report and compared this with structured critical appraisal of the RCT using established tools. We also sought to demonstrate the utility of this novel approach, which we intend to apply in future case studies.

### Identify and analyse articles citing a trial

#### Purposefully select a major surgical RCT

An index RCT was identified and summarised as the case of interest. We sought a highly cited trial report, published in a high-impact journal within the last 10 years. The TIME trial [19], comparing open and minimally invasive surgical access for removal of oesophageal cancer, was selected as it met these criteria and was within our area of expertise.

#### Identify and systematically sample articles citing the RCT

All articles citing this RCT were identified using Web of Science and Scopus citation tracking tools. Letter, editorial and discussion article types were included. On-line comments were identified using the Altmetric.com bookmarklet. Non-English language articles were excluded. Searches were conducted in October 2017.

#### Undertake in-depth qualitative analysis and identify relevant themes

Included articles were thematically analysed using the constant comparison technique, adopted from grounded theory [23, 24]. Articles were read in detail, with no a priori coding framework. Text was considered against the research topic, which focused on understanding how the authors interpreted, appraised and/or applied the findings of the trial. New findings or interpretations were continuously related to existing findings to develop the data set as a whole (i.e. the constant comparison technique). Coding was not constrained by pre-defined boundaries defining relevance. Rather, this was guided by the content of the articles being analysed. During analysis, it transpired that understanding authors’ interpretations of the RCT required examination of their discussion of evidence from other studies. Therefore, other articles cited by the authors were sought to determine the types of evidence being referenced. The designs of these additional studies were ascertained based on the descriptions in those articles (rather than our assessment).

Analysis was performed by BEB and LR. BEB is a senior surgical trainee and postdoctoral researcher with previous experience of qualitative research. LR is a Lecturer in Qualitative Health Science with an interest in trial recruitment issues, implementation of trial evidence, and experience of working on multiple surgical RCTs. Both researchers work within a department with expertise in trials methodology and have detailed knowledge in this field which is likely to have influenced their identification and coding of relevant themes.

Two rounds of double coding of five articles were performed by BEB and LR. Further coding was conducted by BEB and reviewed among the team to revise coded themes. Descriptive data on authorship and origins of the articles were collected.

#### Summarise validity and reporting of the RCT

The RCT was assessed by BEB using a range of critical appraisal tools commonly used to appraise RCTs. These included two of the most commonly used tools to assess RCTs: one examining trial reporting in a broad sense (Consolidated Standards of Reporting Trials for Non-Pharmacological Treatments (CONSORT-NPT) [5]), and another focusing on internal validity as commonly assessed in systematic reviews of trials (the updated Cochrane Risk of Bias Tool (ROBT 2.0) [7]). In addition, the Pragmatic Explanatory Continuum Indicator Scale (PRECIS-2) tool [8] was included, to examine domains associated with the broad applicability and utility of the trial, and the Context and Implementation of Complex Interventions (CICI) framework [25] was included on an exploratory basis to identify broader contextual factors that could be relevant. JMB contributed to assessment during piloting of the tools and in discussion with BEB where there was uncertainty.

#### Broad comparison of all results to develop deeper understanding of how trials are understood and relationship with trial quality

The results of both qualitative analysis and structured critical appraisal were considered side-by-side, with the overall aim of better understanding how other authors’ interpretations of the TIME trial compared with the critical appraisal guided by the above tools. The qualitative analysis of the authors’ interpretations was conducted before the structured critical appraisal to ensure the coding/themes were grounded in authors’ writings, rather than our experience of conducting the structured appraisals. The final step aimed to draw together both analyses, to see whether authors discussing the trial raised concerns across similar domains to the areas covered by the critical appraisal tools, or whether their topics of discussion addressed other considerations.

### Ethical considerations

This study involved secondary use of publicly available written material and did not require ethical review.

### Patient and public involvement

Patients and members of the public were not involved in any aspect of the design of this study.

## Results

### Summary of index RCT

The TIME trial was a two-group, multicentre randomised trial comparing a minimally invasive approach to the surgical removal of oesophageal cancer with an open approach to the abdomen and chest. It was conducted in five centres across four European countries from 2009 to 2011 and is summarised in Table 1.

**Table 1:** Summary of TIME trial

Domain	Description
Population	• Patients with surgically resectable (cT1–3, N0–1, M0) squamous cell carcinoma, adenocarcinoma or undifferentiated carcinoma of the intrathoracic oesophagus or Siewert type 1 oesophago-gastric junction tumours • Underwent neoadjuvant therapy • Age ≥ 18 years and ≤ 75 years • European Clinical Oncology Group performance status 0–2
Intervention	• Minimally Invasive Oesophagectomy (MIO): supine laparoscopic gastric mobilisation and prone thoracoscopic procedure with either cervical or intra-thoracic anastomosis
Comparator	• Open two- or three-phase oesophagectomy with cervical or intra-thoracic anastomosis
Outcome	• 2-week and in-hospital pulmonary infection rates
Results	• 59 patients had MIO and 56 patients underwent open surgery • 9% vs 34% pulmonary infection rate (relative risk 0.30, 95% confidence interval 0.12–0.76, *p* = 0.005)
Conclusion	• This ‘first randomised trial’ comparing MIO and open approach provided ‘evidence for the short-term benefits of minimally invasive oesophagectomy for patients with resectable oesophageal cancer’.

### Characteristics of articles

Searches identified 26 articles, and 23 were included (exclusions: an incorrectly classified case report and two articles in German). Summary characteristics are provided in Table 2. Most articles (18/23, 78%) originated from Europe or the United States. The majority (20/23, 87%) included at least one author holding an academic position; 18/23 (78%) included at least one professor or associate professor (as defined within their own institution). Nearly all included at least one consultant or trainee surgeon (21/23, 91%).

**Table 2:** Summary of characteristics of included articles

		*n* = 23
Article type	Editorial / discussion	13
	Letter	10
Continent of origin	Europe	12
	United States	6
	Asia	4
	Australasia	1
Number of authors	1	10
	2	5
	3	4
	≥4	4
Academic authorship	At least one professor / associate professor	18
	At least one other academic position	2
	No academic authorship	3
Surgeon authorship	At least one consultant surgeon	20
	At least one trainee surgeon	1
	No surgeons	2
Journal impact factor	< 2	1
	2–4.9	11
	5–9.9	1
	10–19.9	0
	≥20	8
	N/A	2
Year of publication	2012	10
	2013	5
	2014	0
	2015	3
	2016	1
	2017	4

Altmetric.com identified several references to the TIME trial, detailed in Table 3. Only one, part of the British Medical Journal blog series, included text discussing the trial, rather than simply restating its results or directing readers to the study report.

**Table 3:** Summary of references to TIME trial identified using Altmetric.com (searched 10 September 2018)

Type	Description	n
Blog	One part of a journal review on a British Medical Journal blog page, summarising problems with TIME trial in one sentence.	1
Policy document	National Institute for Health and Care Excellence National Guideline 83 on assessment and management of oesophago-gastric cancer. National policy document including evidence synthesis of trial results with no specific discussion of TIME trial.	1
Tweets	No discussion of TIME trial. 11 retweets from The Lancet, several others based on same tweet.	24
Facebook pages	1 US cardiothoracic and vascular surgery group and 1 Argentinian medical practice post providing link to trial without comment.	2
Research highlight platform	3 members of F1000Prime recommended this trial.	1

### Themes identified

Qualitative analysis resulted in description of three key themes: identification of wide-ranging issues with the RCT; limited appraisal of non-RCT studies; and variable recommendations for future practice and research. Codes linking quotes to articles and bibliographic data are provided in supplementary Table 1.

#### Identification of wide-ranging issues with the RCT

Authors extensively discussed and critiqued several features of trial design and conduct. These included the population, intervention and outcomes of the trial.

*If the author’s primary outcome was focused on pulmonary infection, perhaps other patient associated inclusion / exclusion criteria may have been of value. These would include patients with poor pulmonary function parameters … patients with major organ disease … and recent history of prior malignancy. (E2).*

*In the present [TIME] trial, the difference between minimally invasive and open oesophagectomy was maximised with a purely thoracoscopic (prone position) and laparoscopic technique. (E1).*

*The primary outcome … was pulmonary infection within the first 2 weeks after surgery and during the whole stay in hospital. This cannot be considered as the relevant primary outcome with reference to the decision problem outline by the authors … (E5).*

Beyond these basic trial design parameters, authors of the citing articles also highlighted important confounding variables.

*Many non-studied variables, including malnutrition, previous and current smoking, pulmonary comorbidities, functional status, and clinical TNM (tumour, node, metastasis) staging, have all been shown to strongly affect the primary endpoint of this trial – postoperative pulmonary infection. (L2).*

*Several correspondents suggest that lower rates of respiratory infection might have been achieved by use of alternative strategies for preoperative preparation, patient positioning, ventilator settings, anaesthetic agents, or postoperative care. (L6).*

The articles also covered other potential problems with the trial, such as sample size and learning curve effects.

*The sample size for sufficient statistical power for major morbidity, survival, total morbidity and other similarly important outcomes may actually be larger. (E2).*

*The inclusion criteria for participating surgeons appears to have the performance of a minimum of only 10 MIOs and this low level of experience may be reflected in relatively high conversion rate of 13%. (E4).*

Only one article (E2) made clear statements praising aspects of the trial:

*‘…The protocols for the RCT appear sound with randomization, intention to treat, PICO … and bias elimination.’*

The next sentence of this article balanced these positive comments with discussion of limits due to the lack of blinding and other potential confounding variables.

#### Limited appraisal of non-RCT studies

Authors often cited other types of evidence in the same field to support their views without discussing their methodological limitations. Types of evidence included single-surgeon series, non-randomised comparative studies, systematic reviews (SRs) and meta-analyses (MAs).

*Luketich* et al.*, one of the earlier pioneers of MIE, reported their extensive experience of 1033 consecutive patients undergoing MIE with acceptable lymph node resection, postoperative outcomes, and a 1.7% mortality rate. (L8).*

*In a population-based national study, … the incidence of pneumonia was 18.6% after open oesophagectomy and 19.9% after minimally invasive oesophagectomy … (L3).*

*Although systematic reviews and a large comparative study of minimally invasive oesophagectomy have not shown this technique to be beneficial as compared with open oesophagectomy, some meta-analyses have suggested specific advantages. (E1).*

The existing SRs and MAs were discussed in relation to the intervention and its outcomes, without directly relating them to the TIME trial itself. The implications for authors’ impressions of the TIME trial findings were generally unclear.

There was limited appraisal of these SRs and MAs, especially when contrasted with discussion of the TIME trial. Several authors referred to the large, single-surgeon series of MIO by Luketich, but only one author described limits of this single-institution non-comparative study.

*We must not rely on the limitation of single-institution studies and historical data. This procedure must be broadly applicable and not the domain of a few experts for it to become the new gold standard. (E12).*

A few others highlighted the limits of other study designs, but there was a striking disparity in the level of critique, when compared with that of the TIME trial.

*In their systematic review … Uttley* et al. *correctly conclude that due to factors such as selection bias, sufficient evidence does not exist to suggest the MIO is either equivalent to or superior to open surgery. (E6).*

*All these studies however, concede that due to a lack of feasible evidence by way of prospective randomized controlled trials (RCT), no definitive statement of MIE ‘superiority’ over standard open techniques can be made. (E2).*

Although several authors referred to the existing SRs and MAs, none reported the design of the included primary studies, which were largely retrospective and non-randomised.

#### Variable recommendations for future practice and research

The authors had differing interpretations and recommendations for implementation based on the TIME trial. Some articles discussed issues with the trial and did not make recommendations for future practice, in some cases asking for additional information to better understand or interpret the trial.(L1, L3–5) For example, one simply wrote that the authors ‘have several concerns’, before reporting differences in outcomes between TIME and other studies, and describing practice in their own institution. (L1) Others reported that more work was required, such as further analysis of long-term results of patients included in TIME, or called for further trials in different patient populations.

*However, the main issue which this study [TIME] does not address is that of long-term survival. … If the authors can indeed demonstrate at least equivalent long-term oncological outcome for MIO and open oesophagectomy, then this paper should provide an impetus for driving forward the widespread adoption of MIO. (E4).*

*Of interest will be whether similar results can be repeated in patients in Asia, with mainly squamous cell cancers that are proximally located. … The substantial benefit shown in this trial [TIME] … might encourage investigators to do further randomised studies at other centres. If these results can be confirmed in other settings, minimally invasive oesophagectomy could truly become the standard of care. (E1).*

One article (E6) considered the evidence for MIO, discussed this against methodological aspects of a colorectal trial evaluating a minimally invasive approach, before restating the findings of TIME, opining that:

*‘This study confirms that RCT [sic] for open versus MIO is indeed possible, but further larger trials are required.’*

Later in that article, the authors suggested extensive control of wide-ranging aspects of perioperative care would be important for future trials.

Authors of three articles (E7, E9, E11) suggested that the available evidence was enough for increasing adoption of MIO.

*…The available evidence increasingly favors a prominent role for minimally invasive approaches in the management of esophageal cancer. Endoscopic therapies and minimally invasive approaches offer at least equivalent oncologic outcomes, with reduced complications and improved quality of life compared with maximal surgery. (E11).*

*We are close to a situation in which one can argue that MIE is ready for prime time in the curative treatment of invasive esophageal cancer. If we critically analyse the level and grading of evidence, the current situation concerning MIE and hybrid MIE is far better than was the case when laparoscopic cholecystectomy, anti-reflux surgery, and bariatric surgery were introduced into clinical practice. (E9).*

No authors called for the cessation of MIO, although one referred to some centres stopping ‘their MIE [minimally invasive esophagectomy] program due to safety reasons’. (E13).

### Assessment of RCT using validated tools

The TIME trial results and protocol papers [19, 26] were examined to assess the trial and its reporting. Assessment using CONSORT-NPT demonstrated reporting shortfalls in several areas (full notes in supplementary Table 2). These included: lack of information on adherence of care providers and patients to the treatment protocol; discrepancies between the primary outcomes proposed in the protocol (3 pulmonary outcomes) and the trial report (one pulmonary result); no information on interim analyses or stopping criteria; a lack of information regarding statistical analysis to allow for clustering of patients by centre; and absence of discussion of the trial limitations or generalisability.

Risk of bias was assessed as shown in Table 4. Overall, the TIME trial was considered at high risk of bias.

**Table 4:** Risk of bias determined using the Cochrane Risk of Bias Tool 2.0

Domain of bias	Bias judgement	Support for judgement
Randomisation	Low	‘When informed consent is obtained, the patient will be randomized at the outpatient clinic. Randomization is performed per center by an internet randomization module maintained by coordinators at the VUmc.’
Deviations from intended interventions	High	‘Patients, and investigators undertaking interventions, assessing outcomes, and analysing data were not masked to group assignment.’ ‘Open oesophagectomy involved … the lateral decubitus position with double tracheal intubation and lung block… Minimally invasive oesophagectomy was performed … in the prone position … with single-lumen tracheal intubation…’
Missing outcome data	Low	All randomised patients included in intention-to-treat analysis.
Measurement of outcome	High	‘Patients, and investigators undertaking interventions, assessing outcomes, and analysing data were not masked to group assignment.’ Imaging and sputum culture decisions made by team providing postoperative care for patient, not blinded to treatment allocation.
Selection of reported result	High	Protocol: ‘The primary endpoint of this study concerns the respiratory complications (i.e. infections) within two weeks after the operation. This is categorized as: grade 1) initial respiratory after operation with continued mechanical ventilation; grade 2) after successful detubation, clinical manifestation of respiratory infection caused by (broncho) pneumonia, confirmed by thorax X-ray or CT scan … and a positive sputum culture; and grade 3) other thoracic infections…’ Report: the primary outcome was postoperative pulmonary infection, defined as clinical manifestation of pneumonia or bronchopneumonia confirmed by thoracic radiographs or CT scan … and a positive sputum culture …’
Overall	High	

Assessment using the PRECIS-2 tool is shown in Table 5. Overall, TIME had features in keeping with a more pragmatic rather than explanatory trial. This suggested a reasonable degree of applicability and usefulness to wider clinical practice.

**Table 5:** Assessment of the TIME trial using the PRECIS-2 tool

Domain	Rating	Support for judgement
Eligibility criteria	3	Included oesophageal and Siewert 1 tumours; pre-operative T1–3, N0–1; aged 18–75 years. Therefore, excluded Siewert 2, pre-operative T4 and N2–3, those aged > 75 years.
Recruitment path	5	‘The patient will be informed about the trial at the outpatient clinic.’
Setting	4	International study across 5 centres in 3 countries in Europe, including academic and non-academic units. Units performing > 20 oesophagectomies per year.
Organisation	3	No extra staffing. Surgeons in some centres had been proctored by lead centre, but not as part of the trial. Surgeons from these centres submitted videos to judge their experience and skill to be allowed to participate. Other centres ‘already well experienced’. Surgeons required a minimum of 10 MIO to participate.
Flex of experimental intervention - delivery	3	Operation was specified in protocol, but not exhaustively. For either open or MIO, the operation could be 2 or 3 stage. There was no monitoring to ensure compliance. Positioning and aspects of anaesthesia were specified, including left decubitus position and right lung block for open thoracotomy, with prone positioning and no lung block for MIO.
Flex of experimental intervention - adherence	–	As per PRECIS-2 toolkit, not applicable when patients undergoing surgery.
Follow-up	5	Follow-up as per usual practice, at 6 weeks, 3 months, 6 months and 1 year, until 5 years postop.
Outcome	3	Primary outcome outlined in the protocol included all pulmonary infections, with 3 categories discussed. However, the trial report only presents one of these 3 categories, pneumonia (CXR/CT changes and positive sputum culture). No patient-centred outcomes, such as Patient Reported Outcome Measures. Outcomes were measured early in the patient’s recovery from the procedure.
Analysis	5	Intention-to-treat analysis of all randomised patients with no apparent loss to follow-up.
Median	3.5	

Application of the CICI framework highlighted several higher-level considerations relevant to the applicability of the TIME trial not described in the protocol or study report (see Table 6). These included lack of detail on the setting, as well as epidemiological and socio-economic information.

**Table 6:** Notes on domains relevant to the implementation of MIO based upon the CICI checklist

Domain	Notes
Setting	Report describes types of hospital, total number of centres and countries participating in the study.
Geographical	Participating countries identified, but no discussion of access to healthcare system in each.
Epidemiological	No discussion of incidence and prevalence of the condition, usual morbidity and mortality rates.
Socio-economic	No discussion of burden of disease or access to the healthcare system.
Socio-cultural	Uncertain relevance of this domain for this study, intervention and the intended audience for the trial.
Political	No discussion of the type of healthcare system, its resources and access.
Legal	No discussion of guidelines outlining the existing role of the intervention.
Ethical	Statement declaring no conflict of interests included.
Provider	Details of the skills, experience and training of participating surgeons was included. No discussion of attitudes towards the intervention or motivation for participating in the trial.
Organisation and structure	No discussion of the size, structure and culture of the participating organisations. However, as a multicentre study, natural variation in these variables will have occurred, improving external validity.
Finance	Funding for the trial was acknowledged. However, there was no discussion of financial incentives, costs or future funding for adoption of the intervention.
Policy	No discussion of the role of evidence-based medicine in determining policy.

Overall, these tools suggested that TIME had several limitations. These included issues with standardisation and monitoring of intervention adherence, lack of blinding, failure to use hierarchical analysis and a lack of information on provider volume. The risk of bias was high, limiting confidence attributing outcomes to the allocated interventions. Broad applicability was considered reasonable, though study utility was compromised by a short-term clinical outcome, rather than longer term or patient-reported outcomes. While TIME may have provided early evidence for benefit of MIO to reduce pulmonary infection within 2 weeks of surgery, the appraisal suggested more evidence was needed before considering wider adoption of MIO.

### Broad comparison of all results to develop deeper understanding

We considered the findings from the qualitative analysis in relation to those of the critical appraisal. In doing so, broad domains of internal and external validity seemed a useful system to bring together results of both analyses. While the ROBT was described by its creators as focused on internal validity, the PRECIS-2 and CICI tools were not described in terms of validity. Rather, their authors referred to applicability and reproducibility in other settings, which may also be described as external validity. CONSORT-NPT is a tool focused on reporting of trials, and its authors referred to both domains, with some duplication of factors covered in the other tools. However, authors of the articles included in the qualitative analysis did not adopt such methodological terminology when expressing concerns about these aspects of the index RCT’s conduct or reporting.

Robust internal validity allows confident attribution of treatment effects to the experimental intervention. The ROBT identified high risk of bias in the TIME trial. Qualitative analysis revealed discussion of various aspects relevant to internal validity. For example, several authors discussed differences in patient positioning and anaesthetic techniques. These confounding variables may have introduced systematic differences in care between groups, aside from the allocated intervention, resulting in bias. However, the article authors did not articulate the implications of their concerns in such terms and did not consider whether these problems rendered the trial fatally biased.

Sound external validity suggests similar treatment effects may be achieved by other clinicians in other settings for other patients. Pragmatic trials have broad applicability, with wide inclusion criteria, and patient-centred outcomes. The PRECIS-2 describes domains relevant to this applicability. TIME had several features of a pragmatic trial, suggesting relatively broad applicability. The qualitative analysis showed authors were concerned about these issues. For example, several discussed the appropriateness and utility of 2-week and in-hospital pulmonary infection rates as the primary outcome measure. However, authors did not directly relate such concerns to external validity or generalisability, to reach a conclusion about whether the trial should influence practice.

While many authors identified issues relevant to internal and external validity, the lack of clear explanation of their implications meant it was difficult to determine whether they thought the trial justified a change in practice. This contrasts with the structured assessments, which defined clear problems with the trial and limits to its usefulness.

## Discussion

This study presents the first application and results of a new method to generate insights into how evidence from a trial was understood, contextualised and related to practice. Qualitative analysis of letters and editorials, largely written by academic surgeons, documented extensive discussion of problems with the trial, but without clear formulation of the implications of these concerns for its internal or external validity and applicability. These authors reached a variety of conclusions about the implications of the trial for surgical practice. A separate assessment using structured tools defined specific weaknesses in trial methodology. Whilst this new approach yielded useful findings in this single case study, the method should be further tested using multiple trials and cross-case analyses. The initial findings based on this single case study suggest a need to clarify standards against which a trial may be assessed to guide decisions about its role in changing practice, and potentially also to guide efforts to influence practitioners to implement change if appropriate. Within this, our findings suggest a need to focus efforts on educating surgeons about trial design and quality, which may contribute to implementation science-based efforts to inform clinical decision-making and implementation of trial results.

This study contributes to the wider literature showing that evidence does not speak for itself. New evidence is often considered alongside competing bodies of existing evidence that may support different ideas, theories or interventions [27, 28]. When a study is published, this new evidence is assimilated into the wider scientific context. Its strengths, weaknesses and overall contribution are debated and disputed. Through the lens of Latour’s actor-network theory [29, 30], the new trial can be considered a novel actor within the wider network of actors that includes other trials and studies of the intervention, as well as the consumers of this evidence. Those commenting on the trial have an important role in how different features of the trial are identified, discussed and debated, and how its findings are framed. This agency may be influenced by their own clinical experience, education, skill set, work environment and colleagues, amongst other factors. Given these complexities, it is not surprising to find that different authors reached different conclusions about the TIME trial.

The way authors of the included articles used and appraised different types of study raises questions about how the hierarchy of evidence, and the primacy of the RCT, is applied to routine clinical practice. We found extensive criticism of the TIME trial. Article authors described several limitations relating to its population, intervention, associated co-interventions and confounding variables, as well as the outcomes selected. Certainly, the authors presented valid criticisms that limited the trial’s validity, as identified by structured critical appraisal. Over recent years, trials methodologists have worked to better understand and optimise many such aspects of trial conduct. The development of the CONSORT reporting standards promotes detailed description of key methods, such as random sequence generation and allocation concealment, that allow critical judgements about internal validity to be made [5]. The growth of pragmatic trials, featuring wide inclusion criteria, conducted across multiple sites, with clinically meaningful outcomes, reflects a concerted effort to improve applicability or external validity of RCTs [8, 31]. It may never be possible to conduct a ‘perfect’ trial, but improvements in the rigor and transparency of design hopefully ensure that RCTs can provide sufficiently robust evidence that is useful to the broad population of patients and clinicians within a healthcare system. Whether these developments, designed to address valid criticisms of RCTs, are widely understood outside the sphere of trials methodologists is unclear.

Conversely, the authors of the included articles were far less critical of non-RCT evidence. For example, several authors referred to the single-surgeon case-series of Luketich [32]. Only one author discussed its limitations for generalisation. Surgical skill and performance vary [33]; what is *possible* for a single surgeon cannot be generalised to what is *usual* for most. Similarly, authors cited systematic reviews and meta-analyses without clear description of the original study designs. Evidence synthesis cannot eliminate biases in retrospective, non-randomised studies using statistical techniques. Failure to clearly articulate limitations of these different studies may support our contention that the authors lacked appropriate appraisal skills. Alternatively, it may suggest bias in favour of the intervention, such that the authors understood, but did not want to articulate its limitations.

While RCTs have not been toppled from their position at the top of the hierarchy of evidence about the efficacy of interventions, developments in other areas have seen increasingly sophisticated use of observational data to better understand the effects of treatments. Researchers have taken advantage of increasing availability of vast quantities of genetic data. In epidemiology, the concept of Mendelian randomisation has been used to try and unpick causal relationships from non-causative correlations [34]. At the patient level, genetic testing of different types of cancer has allowed targeting of treatments according to cellular sensitivities [35]. The development of such markers by which to tailor treatment have led to proposals of an idealised future whereby individual treatments are entirely personalised according to a panel of markers that accurately predict treatment response and prognosis. These different research approaches are inevitably competing for resources and intellectual priority. However, as has been argued by Backmann, for these other study types to take priority, “what needs to be shown is not only that RCTs might be problematic …, but that other methods such as cohort studies actually have better external validity.” [36]

Evidence-based medicine aims to apply the best available evidence to individual patients [37]. This aim, by its very nature, creates a disconnect between evidence from RCTs, which are aggregated studies of groups of patients to determine average effects, and clinical decision-making at the individual level [38]. This could be considered to represent an insurmountable ‘get-out’ clause, whereby a clinician may always justify deviation from ‘the evidence’ due to differences between the patient in front of them and those included in the relevant study. It may also prove very difficult to allow the theory-based weight of a journal article to over-ride an individual clinician’s personal lived experience of different interventions and their efficacy. This may be particularly problematic in surgical practice [16] where the practitioner is usually physically connected with the intervention. This may increase the importance attached to experience, even if that experience is at odds with large-scale studies. We do not disagree that clinicians must treat individual patients according to their specific condition and their wishes. However, it may be considered that aggregate practice, across a surgeon’s cases or across a department, should fall roughly in line with an appropriate body of suitably valid and relevant evidence.

Implementation science research has illuminated many factors affecting implementation beyond knowledge of the evidence. Damschroder et al. described the Consolidated Framework for Implementation Research (CFIR) to identify real-world constructs influencing implementation, relating to the intervention, individuals, organisations and systems [39]. These included ‘evidence strength and quality’ as well as ‘knowledge and beliefs about the intervention’, constructs readily identified within the present study. Their framework also highlights many other important factors such as cost, patient needs and resources, peer pressure, external policies and incentives, and organisational culture. Surgical research has demonstrated wide variation in practice, even in the presence of high quality evidence [40], and the broad range of factors affecting implementation of interventions, such as Enhanced Recovery After Surgery [41]. Our approach may contribute as another tool to understand barriers and facilitators to evidence implementation. It may prove particularly useful in conjunction with other methods such as interviews and observations, informed by a relevant framework, such as the Theoretical Domains Framework [42, 43].

The early promise of our new method needs further work to conduct multiple case studies of different RCTs to allow cross-case analyses and a more thorough understanding of how RCTs are interpreted and appraised in the landscape of written commentaries. Examination of further case-studies may also inform refinements to the methods. For example, further analyses may indicate recurring themes across case-studies, which may in turn contribute towards a priori coding criteria and more efficient approaches to analyses (e.g. framework analysis [44]). It will also be important to include assessment of how each trial is situated in the wider context of relevant evidence, across study types. For individual trials, combined qualitative and structured analyses may determine the extent to which that RCT is flawed and requires further evaluation in a more methodologically sound study. Alternatively, it may demonstrate that the problem in bridging the gap between evidence and practice resides in the competition between different bodies of evidence, comprised of different types of study, and appropriate understanding of their strengths and weaknesses, as well as their applicability to practice. Work should also be undertaken to investigate how contemporary practice may have changed alongside publication of such articles, to investigate the relationship between what is written about the trial, and clinical practice as delivered.

While this study has shown the potential of this new method, its strengths and limitations must be considered. Rigorous analysis using robust qualitative methods and double coding by experienced researchers was undertaken. The articles examined were written without knowledge that they would be analysed in this manner, limiting bias this could introduce. The use of multiple tools to assess the index RCT created a broad overview of its strengths and weaknesses. The most important study limitation was that we did not directly explore authors’ understandings and interpretations, so underlying understanding of the key issues was inferred, rather than directly scrutinised. Failure to articulate is not the same as a lack of understanding. Further, we did not ask authors their motivations to publish their articles, an activity with its own significance. In addition, this study attempted to provide insights into the authors understanding and interpretation of the trial, and it does not purport to be an assessment of practice itself, which would benefit from other approaches to investigation (e.g. qualitative observations, interviews, quantitative procedure rate analyses). This study applied our new method to a single, surgical RCT. The issues identified may be particular to that intervention, specialty, or trial design; further case studies are required to determine broader relevance.

## Conclusions

This study has successfully applied a new method to better understand how clinicians and academics understand evidence from a surgical RCT - the TIME trial. It identified discussion of many issues with the trial, but the authors who cited the trial did not specifically articulate the implications of these issues in terms of its internal and external validity. The authors reached a wide range of conclusions, ranging from further evaluation of the intervention, to widespread adoption. Structured appraisal of TIME suggested that the trial was at high risk of bias with limited generalisability. Further application of this method to multiple trials will allow cross-case analyses to determine whether the issues identified are similar across other trials and yield information to better understand how this type of evidence is interpreted and related to practice. This approach may be complemented by other data, such as in-depth interviews. This may reveal genuine flaws in trial design that limit application, or that other issues such as poor understanding or competing non-clinical factors impede the translation of evidence into practice. We hope that this work may help existing efforts to close the research-practice gap, and help ensure that patients receive the best care, based upon the highest level of evidence.

## Supplementary information

**Additional file 1 Table S1.** Identifying codes and bibliographic information on all citing articles included in analysis. **Table S2.** CONSORT-NPT checklist with notes on TIME trial.

## Data Availability

The dataset upon which this work is based consists of articles already available within the published literature.

## Abbreviations

RCT: Randomised controlled trial
TIME: Traditional Invasive versus Minimally invasive Esophagectomy
CONSORT-NPT: CONsolidated Standards Of Reporting Trials for Non-Pharmacological Treatments
PRECIS: PRagmatic Explanatory Continuum Indicator Scale
CICI: Context and Implementation of Complex Interventions
ROBT: Risk Of Bias Tool
MIO: Minimally Invasive Oesophagectomy
SR: Systematic Review
MA: Meta-Analysis
