# Supplementary material for: A mixed methods case study investigating how randomised controlled trials (RCTs) are reported, understood and interpreted in practice
Source: BMC Med Res Methodol. 2020 May 12;20:112. doi: 10.1186/s12874-020-01009-8 (PMC7216481; doi:10.1186/s12874-020-01009-8)
Supplement: Supplementary file 1 — Additional file 1 Table S1. Identifying codes and bibliographic information on all citing articles included in analysis. Table S2. CONSORT-NPT checklist with notes on TIME trial. [file 12874_2020_1009_MOESM1_ESM.docx]

| **ID** | **Type** | **Authors** | **Title** | **Journal** | **Year** | **Vol** | **Iss** | **Start p** | **End p** | **PubMed ID** |
| --- | --- | --- | --- | --- | --- | --- | --- | --- | --- | --- |
| E1 | Editorial | Law, S | Is minimally invasive preferable to open oesophagectomy? | Lancet | 2012 | 379 | 9829 | 1856 | 1858 | 22552193 |
| E2 | Editorial | Dantoc, MM; Cox, MR; Eslick, GD | The first randomised controlled trial on minimally invasive esophagectomy (MIE) and the ongoing quest for greater evidence | Journal of Thoracic Disease | 2012 | 4 | 5 | 459 | 461 | 23050107 |
| E3 | Editorial | Cuesta, MA; Biere, SSAY; Henegouwen, MIV; van der Peet, DL | Randomised trial, Minimally Invasive Oesophagectomy versus open oesophagectomy for patients with resectable oesophageal cancer | Journal of Thoracic Disease | 2012 | 4 | 5 | 462 | 464 | 23050108 |
| E4 | Editorial | Khan, O; Nizar, S; Vasilikostas, G; Wan, A | Minimally invasive versus open oesophagectomy for patients with oesophageal cancer: a multicentre, open-label, randomised controlled trial | Journal of Thoracic Disease | 2012 | 4 | 5 | 465 | 466 | 23050109 |
| E5 | Editorial | Uttley, L; Campbell, F; Rhodes, M; Cantrell, A; Stegenga, H; Lloyd-Jones, M | Minimally invasive esophagectomy versus open surgery: is there an advantage? | Surgical Endoscopy | 2013 | 27 | 11 | 4401 | 4402 | 23807755 |
| E6 | Editorial | Hamilton, E; Griffiths, EA | Commentary on "Uttley L, Campbell F, Rhodes M et al. Minimally invasive esophagectomy versus open surgery: is there an advantage? Surg Endosc 2013;27(3):724-731" | Surgical Endoscopy and Other Interventional Techniques | 2013 | 27 | 11 | 4399 | 4400 | 23820867 |
| E7 | Editorial | Weijs, TJ; Ruurda, JP; Nieuwenhuijzen, GAP; van Hillegersberg, R; Luyer, MDP | Strategies to reduce pulmonary complications after esophagectomy | World Journal of Gastroenterology | 2013 | 19 | 39 | 6509 | 6514 | 24151374 |
| E8 | Editorial | Harisch, K | Current and evolving surgical strategies in thoracic oncology | Indian Journal of Surgical Oncology | 2013 | 4 | 2 | 94 | 95 | 24426707 |
| E9 | Editorial | Lundell, L | Quality of Life after Minimally Invasive Versus Open Esophagectomy | World Journal of Surgery | 2015 | 39 | 9 | 2109 | 2110 | 24952080 |
| E10 | Editorial | Edil, BH; Schulick, RD | Challenges of Minimally Invasive Pancreas Surgery Growth With Such a High Learning Curve | JAMA Surgery | 2015 | 150 | 5 | 423 | 423 | 25760810 |
| E11 | Editorial | Nason, KS | Minimal or maximal surgery for esophageal cancer? | Journal of Thoracic and Cardiovascular Surgery | 2016 | 151 | 3 | 633 | 635 | 26541830 |
| E12 | Editorial | Thomas, Ng | With minimally invasive esophagectomy, thoracic surgeons must avoid falling into the same trap again! | Seminars in Thoracic and Cardiovascular Surgery | 2015 | 27 | 2 | 216 | 217 | 26689449 |
| E13 | Editorial | Schaap, DP; Nieuwenhuijzen, GA; Luyer, MD | The use of near-infrared fluorescence imaging in the surgical treatment of esophageal cancer | Journal of Thoracic Disease | 2017 | 9 | 2 | 240 | 243 | 28275469 |
| E14 | Editorial | Lee, F; Sarkaria, IS; Luketich, JD | Surgeon proficiency and outcomes in esophagectomy: a perspective and comment on an analysis of the Swedish Cancer Registry | Journal of Thoracic Disease | 2017 | 9 | 3 | E279 | E281 | 28449520 |
| L1 | Letter | Swisher, S; Ajani, J; Correa, A; Komaki, R; Hofstetter, W | Minimally invasive versus open oesophagectomy for oesophageal cancer | Lancet | 2012 | 380 | 9845 | 883 | 883 | 22959377 |
| L2 | Letter | Mariette, C; Robb, WB | Minimally invasive versus open oesophagectomy for oesophageal cancer | Lancet | 2012 | 380 | 9845 | 883 | 883 | 22959378 |
| L3 | Letter | Fujita, T | Minimally invasive versus open oesophagectomy for oesophageal cancer | Lancet | 2012 | 380 | 9845 | 884 | 884 | 22959379 |
| L4 | Letter | Parotto, M; Valenza, F; Ori, C; Spieth, PM | Minimally invasive versus open oesophagectomy for oesophageal cancer | Lancet | 2012 | 380 | 9845 | 884 | 884 | 22959380 |
| L5 | Letter | Spengler, CM; Verges, S; Walder, B | Minimally invasive versus open oesophagectomy for oesophageal cancer | Lancet | 2012 | 380 | 9845 | 885 | 885 | 22959381 |
| L6 | Letter | Avery, KN; Barham, CP; Berrisford, R; et al | Understanding surgical interventions in RCTs: the need for better methodology | Lancet | 2013 | 381 | 9860 | 27 | 28 | 23290962 |
| L7 | Letter | Adenis, A; Robb, WB; Mariette, C | Esophageal Carcinoma | New England Journal of Medicine | 2015 | 372 | 15 | 1471 | 1471 | 25853759 |
| L8 | Letter | Ben-David, K; Hochwald, SN | Letter to the Editor Regarding Wiesel et al.: Minimally Invasive Esophagectomy | Journal of Laparoendoscopic and Advanced Surgical Techniques | 2017 | 27 | 2 | 170 | 170 | 27893302 |
| L9 | Letter | Sawant, A; Mankar, H; Chatterjee, A; Pramesh, CS | VATS Versus Open Lobectomy: Need for a Prospective Trial | Annals of Thoracic Surgery | 2017 | 103 | 2 | 690 | 691 | 28109362 |

Supplementary Table 1. Identifying codes and bibliographic information on all citing articles included in analysis.

| Section/Topic Item | Checklist item no. | CONSORT item | Extension for NPT trials | Notes |
| --- | --- | --- | --- | --- |
| Title and abstract |  |  |  |  |
|  | 1a | Identification as a randomized trial in the title |  | Yes |
|  | 1b | Structured summary of trial design, methods, results, and conclusions (for specific guidance see CONSORT for abstracts) | *Refer to CONSORT extension for abstracts for NPT trials* | Yes |
| Introduction |  |  |  |  |
| Background and objectives | 2a | Scientific background and explanation of rationale |  | Yes |
|  | 2b | Specific objectives or hypotheses |  | Yes |
| Methods |  |  |  |  |
| Trial design | 3a | Description of trial design (such as parallel, factorial) including allocation ratio | When applicable, how care providers were allocated to each trial group | Yes. 1:1 stratified by centre. |
|  | 3b | Important changes to methods after trial commencement (such as eligibility criteria), with reasons |  | No comments |
| Participants | 4a | Eligibility criteria for participants | When applicable, eligibility criteria for centers and for *care providers* | Yes – patients, surgeons and centres. |
|  | 4b | Settings and locations where the data were collected |  | Centres – cities and countries. |
| Interventions*†* | 5 | The interventions for each group with sufficient details to allow replication, including how and when they were actually administered | Precise details of both the experimental treatment and comparator | Yes – reasonable detail in report and protocol |
|  | 5a |  | Description of the different components of the interventions and, when applicable, description of the procedure for tailoring the interventions to individual participants. | No tailoring for individual participants discussed. |
|  | 5b |  | Details *of whether and* how the interventions were standardized. | Limited info – locally more standardized (protocol – videos), other centres less clear – ‘discussed op technique…’ |
|  | 5c. |  | Details *of whether and* how adherence of care providers to the protocol was assessed or enhanced | Not discussed |
|  | 5d |  | *Details of whether and how adherence of participants to interventions was assessed or enhanced* | Not relevant for surgical RCT |
| Outcomes | 6a | Completely defined pre-specified primary and secondary outcome measures, including how and when they were assessed |  | Discussed, but 3 mentioned in protocol, only 1 in results paper. |
|  | 6b | Any changes to trial outcomes after the trial commenced, with reasons |  | Not mentioned |
| Sample size | 7a | How sample size was determined | When applicable, details of whether and how the clustering by care providers or centers was addressed | Provided using local data, though that suggested >50% pneumonia in open. Clustering not discussed. |
|  | 7b | When applicable, explanation of any interim analyses and stopping guidelines |  | None provided |
| Randomization: |  |  |  |  |
| - Sequence generation | 8a | Method used to generate the random allocation sequence |  | Yes |
|  | 8b | Type of randomization; details of any restriction (such as blocking and block size) |  | 1:1 stratified by centre; blocks not mentioned |
| - Allocation concealment mechanism | 9 | Mechanism used to implement the random allocation sequence (such as sequentially numbered containers), describing any steps taken to conceal the sequence until interventions were assigned |  | Not explicit, but central internet randomization at sponsoring centre. |
| - Implementation | 10 | Who generated the random allocation sequence, who enrolled participants, and who assigned participants to interventions |  | Who did these not reported. |
| Blinding | 11a | If done, who was blinded after assignment to interventions (for example, participants, care providers, those assessing outcomes) and how | ~~Whether or not those administering co-interventions were blinded to group assignment~~  If done, who was blinded after assignment to interventions (e.g., participants, care providers, *those administering co-interventions,* those assessing outcomes) and how | No blinding of any parties. |
|  | 11b | If relevant, description of the similarity of interventions | ~~If blinded, method of blinding and description of the similarity of interventions~~ | No specific description of similarity |
|  | 11c |  | *If blinding was not possible, description of any attempts to limit bias* | ‘To prevent surgeon bias, … experienced in open resection … and extensive experience in minimally invasive …’ |
| Statistical methods | 12a | Statistical methods used to compare groups for primary and secondary outcomes | When applicable, details of whether and how the clustering by care providers or centers was addressed | Provided. No mention of hierarchical analysis. |
|  | 12b | Methods for additional analyses, such as subgroup analyses and adjusted analyses |  | None performed. |
| Results |  |  |  |  |
| Participant flow (a diagram is strongly recommended) | 13a | For each group, the numbers of participants who were randomly assigned, received intended treatment, and were analyzed for the primary outcome | The number of care providers or centers performing the intervention in each group and the number of patients treated by each care provider or in each center | Overall flow diagram, but no information on numbers treated by each centre |
|  | 13b | For each group, losses and exclusions after randomization, together with reasons |  | Provided |
|  | 13c |  | *For each group, the delay between randomization and the initiation of the intervention* | Not provided. |
|  | new |  | Details of the experimental treatment and comparator as they were implemented | How implemented not mentioned. |
| Recruitment | 14a | Dates defining the periods of recruitment and follow-up |  | Yes |
|  | 14b | Why the trial ended or was stopped |  | Met recruitment. |
| Baseline data | 15 | A table showing baseline demographic and clinical characteristics for each group | When applicable, a description of care providers (case volume, qualification, expertise, etc.) and centers (volume) in each group. | Yes patients; no centre volume / expertise specifically, except inc criteria. |
| Numbers analyzed | 16 | For each group, number of participants (denominator) included in each analysis and whether the analysis was by original assigned groups |  | Yes |
| Outcomes and estimation | 17a | For each primary and secondary outcome, results for each group, and the estimated effect size and its precision (such as 95% confidence interval) |  | Yes – OR for primary; no OR for secondary |
|  | 17b | For binary outcomes, presentation of both absolute and relative effect sizes is recommended |  | As above |
| Ancillary analyses | 18 | Results of any other analyses performed, including subgroup analyses and adjusted analyses, distinguishing pre-specified from exploratory |  | None |
| Harms | 19 | All important harms or unintended effects in each group (for specific guidance see CONSORT for harms) |  | No unexpected harms – deaths/complications as expected for operation. |
| **Discussion** |  |  |  |  |
| Limitations | 20 | Trial limitations, addressing sources of potential bias, imprecision, and, if relevant, multiplicity of analyses | In addition, take into account the choice of the comparator, lack of or partial blinding, and unequal expertise of care providers or centers in each group | None discussed |
| Generalizability | 21 | Generalizability (external validity, applicability) of the trial findings | Generalizability (external validity) of the trial findings according to the intervention, comparators, patients, and care providers and centers involved in the trial | Not discussed |
| Interpretation | 22 | Interpretation consistent with results, balancing benefits and harms, and considering other relevant evidence |  | Other relevant evidence and potential explanations for results discussed. |
| Other information |  |  |  |  |
| Registration | 23 | Registration number and name of trial registry |  | Yes |
| Protocol | 24 | Where the full trial protocol can be accessed, if available |  | Yes |
| Funding | 25 | Sources of funding and other support (such as supply of drugs), role of funders |  | Yes |

Supplementary Table 2. CONSORT-NPT checklist with notes on TIME trial.
